# Randomized trial of neoadjuvant chemotherapy in oropharyngeal carcinoma

**DOI:** 10.1054/bjoc.2000.1512

**Published:** 2000-12

**Authors:** C Domenge, C Hill, J L Lefebvre, D De Raucourt, B Rhein, P Wibault, P Marandas, B Coche-Dequeant, M Stromboni-Luboinski, H Sancho-Garnier, B Luboinski

**Affiliations:** Département de chirurgie cervico-faciale, Institut Gustave Roussy, Villejuif, 94805, France; Département de radiothérapie, Institut Gustave Roussy, Villejuif, 94805, France; Département de Biostatistique et d'Epidémiologie, Institut Gustave Roussy, Villejuif, 94805, France; Département de Chirurgie cervico-faciale, Centre Oscar Lambret, Lille, 59000, France; Département de Radiothérapie, Centre Oscar Lambret, Lille, 59000, France; Département de Cancérologie cervico-faciale, Centre Antoine Baclesse, Caen, 14000, France; Service de Radiothérapie, Centre Hospitalier regional Universitaire, Limoges, 87042, France

**Keywords:** oropharyngeal cancer, chemotherapy, randomized trial, cisplatin, fluorouracil

## Abstract

The objective of the study was to evaluate the effect of neoadjuvant chemotherapy on the survival of patients with oropharyngeal cancer. Patients with a squamous cell carcinoma of the oropharynx for whom curative radiotherapy or surgery was considered feasible were entered in a multicentric randomized trial comparing neoadjuvant chemotherapy followed by loco-regional treatment to the same loco-regional treatment without chemotherapy. The loco-regional treatment consisted either of surgery plus radiotherapy or of radiotherapy alone. Three cycles of chemotherapy consisting of Cisplatin (100 mg/m^2^) on day 1 followed by a 24-hour i.v. infusion of fluorouracil (1000 mg/m^2^/day) for 5 days were delivered every 21 days. 2–3 weeks after the end of chemotherapy, local treatment was performed. The trial was conducted by the Groupe d'Etude des Tumeurs de la Tête Et du Cou (GETTEC). A total of 318 patients were enrolled in the study between 1986 and 1992. Overall survival was significantly better (*P* = 0.03) in the neoadjuvant chemotherapy group than in the control group, with a median survival of 5.1 years versus 3.3 years in the no chemotherapy group. The effect of neoadjuvant chemotherapy on event-free survival was smaller and of borderline significance (*P* = 0.11). Stratification of the results on the type of local treatment, surgery plus radiotherapy or radiotherapy alone, did not reveal any heterogeneity in the effect of chemotherapy. © 2000 Cancer Research Campaign http://www.bjcancer.com


*British Journal of Cancer* (2000) **83**(12), 1594–1598
© 2000 Cancer Research Campaign
doi: 10.1054/ bjoc.2000.1512, available online at http://www.idealibrary.com on IDEAL®

http://www.bjcancer.com

### Randomized trial of neoadjuvant chemotherapy in oropharyngeal carcinoma

**C Domenge[1], C Hill[3], JL Lefebvre[4], D De Raucourt[6], B Rhein[7], P Wibault[2], P Marandas[1], B Coche-Dequeant[5], M Stromboni-Luboinski[3], H Sancho-Garnier[3] and B Luboinski[1] for the French Groupe d'Etude des Tumeurs de la Tête Et du Cou (GETTEC)**

[1]Département de chirurgie cervico-faciale, Institut Gustave Roussy, 94805 Villejuif, France; [2]Département de radiothérapie, Institut Gustave Roussy, 94805 Villejuif, France; [3]Département de Biostatistique et d'Epidémiologie, Institut Gustave Roussy, 94805 Villejuif, France; [4]Département de Chirurgie cervico-faciale, Centre Oscar Lambret, 59000 Lille, France; [5]Département de Radiothérapie, Centre Oscar Lambret, 59000 Lille, France; [6]Département de Cancérologie cervico-faciale, Centre Antoine Baclesse, 14000 Caen, France; [7]Service de Radiothérapie, Centre Hospitalier regional Universitaire, 87042 Limoges, France

**Summary** The objective of the study was to evaluate the effect of neoadjuvant chemotherapy on the survival of patients with oropharyngeal cancer. Patients with a squamous cell carcinoma of the oropharynx for whom curative radiotherapy or surgery was considered feasible were entered in a multicentric randomized trial comparing neoadjuvant chemotherapy followed by loco-regional treatment to the same loco-regional treatment without chemotherapy. The loco-regional treatment consisted either of surgery plus radiotherapy or of radiotherapy alone. Three cycles of chemotherapy consisting of Cisplatin (100 mg/m$^2$) on day 1 followed by a 24-hour i.v. infusion of fluorouracil (1000 mg/m$^2$/day) for 5 days were delivered every 21 days. 2–3 weeks after the end of chemotherapy, local treatment was performed. The trial was conducted by the Groupe d'Etude des Tumeurs de la Tête Et du Cou (GETTEC). A total of 318 patients were enrolled in the study between 1986 and 1992. Overall survival was significantly better ($P$ = 0.03) in the neoadjuvant chemotherapy group than in the control group, with a median survival of 5.1 years versus 3.3 years in the no chemotherapy group. The effect of neoadjuvant chemotherapy on event-free survival was smaller and of borderline significance ($P$ = 0.11). Stratification of the results on the type of local treatment, surgery plus radiotherapy or terogeneity in the effect of chemotherapy. © 2000 Cancer Research Campaign http://www.bjcancer.com

**Keywords**: oropharyngeal cancer; chemotherapy; randomized trial; cisplatin; fluorouracil

Patients with head and neck cancer represent 11% of the malignant tumours diagnosed in French males (Ménégoz et al, 1998). While most small tumours can be cured, a majority of the tumours are advanced and despite progress in surgery and radiotherapy, the prognosis remains poor. In the past years, systemic chemotherapy has been used in an attempt to improve the results of loco-regional treatment. Clinical trials have shown a high response rate with a combination of Cisplatinum (CDDP) and 5 Fluoro-Uracil (5FU) in head and neck carcinoma sites (Decker et al, 1983; Weaver et al, 1985; Calais et al, 1988).

In 1986, we decided to investigate the efficiency of neoadjuvant chemotherapy in a single tumour site: the oropharynx with survival as the main study end-point, and event-free survival as a secondary endpoint.

### MATERIALS AND METHODS

All patients with a biopsy-proven squamous cell carcinoma of all sites of the oropharynx except for the posterior wall and the anterior surface of epiglottis, classified as T2 to T4, $N_0$ to $N_{2b}$, and $M_0$ (UICC-AJC, 1986) were eligible if curative treatment, either radiotherapy or surgery, was considered feasible. Patients older than 69, younger than 18 or in whom chemotherapy was contraindicated, or who had previously been treated, or with multiple tumours were excluded. Patients were randomized by telephone to receive either neoadjuvant chemotherapy or no chemotherapy. Randomization was stratified on the centre and on the local treatment which was either surgery followed or not by radiotherapy, or radiotherapy alone.

The treatment planned consisted, in the study arm, of 3 chemotherapy cycles followed, if possible, by surgery and systematic radiotherapy, or if surgery was impossible by radiotherapy alone. Post-randomization consent was obtained from each patient in accordance with the law in use in France at the time of the trial.

A total of 318 patients treated in 6 centres were included in the trial between 1986 and 1992.

*Received 15 December 1999*
*Revised 1 July 2000*
*Accepted 15 August 2000*

*Correspondence to:* C Domenge, C Hill

### Chemotherapy

Chemotherapy consisted of Cisplatin (100 mg/m$^2$) given in a 1-hour i.v. infusion on day 1 followed by a 24-hour i.v. infusion of fluorouracil (1000 mg/m$^2$/day) for 5 days. This treatment was repeated on day 22 unless tumour progression exceeded 25%, and repeated on day 43 only if tumour regression had been observed. 2–3 weeks after the end of chemotherapy, local treatment (surgery and/or radiotherapy) was performed.

### Surgery

The operative procedure in both groups consisted of a commando procedure i.e. oropharyngeal composite resection with segmental mandibular resection. Resection was performed according to the initial volume before chemotherapy whatever the response. In the same time $N_0$ patients and patients with nodes measuring less than 3 cm underwent modified neck dissection. Patients with nodes exceeding 3 cm or more underwent homolateral radical neck dissection. In the chemotherapy group, surgery was performed 2 to 3 weeks after the end of the last chemotherapy cycle.

### Post-operative radiation therapy

Radiotherapy was delivered by $^{60}$Co γ rays in daily fractions of 2 Gy. In patients with free surgical margins, 50 Gy were delivered over 5 weeks to the tumour site and 50 Gy to the bilateral superior and inferior cervical areas, with a boost of 15 Gy in case of extra-capsular spread. In patients with positive surgical margins, 65 Gy were delivered in 6 $^{1}/_{2}$ weeks to the tumour site and bilateral superior cervical areas, and 50 Gy to the inferior cervical areas, with a boost of 15 Gy in case of extra-capsular spread.

In all cases the posterior spinal area was treated with 42 Gy ($^{60}$Co) completed with 8 Gy by electron therapy and a boost of 15 Gy, in case of extracapsular spread. Radiotherapy was to begin within 10 weeks after surgery.

### Radiotherapy alone

Radiotherapy began 2 to 3 weeks after the completion of chemotherapy. 70 Gy were delivered in 7 weeks to the tumour and bilateral superior cervical nodes, and 42 Gy to the posterior cervical field completed with 8 Gy by electron therapy if nodes were not palpable. At the end of radiotherapy, 5 additional Gy were delivered to the tumour site, in case of residual tumour. A boost of 23 Gy was delivered to palpable posterior cervical nodes by electron therapy. 50 Gy were delivered in 5 weeks to the median and inferior cervical areas, with a boost of 15 Gy to palpable nodes with an electron beam.

### Endpoints

Toxicity was evaluated using WHO scale. Response was assessed by clinical examination at the beginning of each chemotherapy cycle. 7–10 days after the third cycle of chemotherapy, clinical examination and a CT scan were performed. Survival was the primary endpoint. Disease-free survival was a secondary endpoint. Overall survival was to be calculated as the time from the date of randomization to either the date of death or to the date of the last follow-up. Event-free survival was to be calculated as the time from the date of randomization to the date of the first event which was documented as a recurrence (local, loco-regional or metastatic), or a second primary, or death, or to the date of the last follow-up.

### Statistical analysis

A total of 760 patients were to be included in the trial, 400 in the surgery followed by radiotherapy stratum and 360 in the radiotherapy alone stratum. These sample sizes allowed a 90% chance of detecting a difference of 10% in the survival rate and 3 analyses were to be conducted after 18, 36 and 60 months of follow-up ($\alpha = 5\%$).

The analysis was to include all randomized patients and use the intention-to-treat principle.

Survival curves used Kaplan–Meier plots, and were to be compared using the log-rank test. The median follow-up time was computed using the inverse Kaplan–Meier method (Schemper and Smith, 1996). A marginal hazards Cox model (Wei et al, 1989) has been used to estimate simultaneously the effects of chemotherapy on the risk of first local recurrence or head and neck second primary, first metastasis, first second primary other than head and neck and death. This model takes into account the correlation between events in the same patient.

### RESULTS

This trial was activated in 1986 and closed to patient accrual in 1992. A total of 318 patients were included in the study, 157 in the induction chemotherapy group and 161 in the no chemotherapy group. The trial was interrupted after 6 years of accrual, because the accrual rate had become very low.

Patient characteristics are presented in Table 1. They were well balanced and there were no significant differences between the treatment arms with respect to sex, age, primary site, T, N stage and performance status. Table 2 gives the TNM distribution by treatment group.

### Treatment

*Surgery*

Among the 71 patients in the chemotherapy group, and for whom surgery had been planned, 11 did not undergo surgery: one patient died of heart failure between chemotherapy cycles and radiotherapy was preferred for 10 others, 5 as they were considered good responders and 5 as they refused surgery. Among the 73 patients who were not to receive chemotherapy and for whom

**Table 1** Patient characteristics

| Characteristic | Treatment group: No chemotherapy (161) | | Treatment group: Chemotherapy (157) | |
|---|---|---|---|---|
| Age: mean (SD[a]) | 54.2 | (8.3) | 52.4 | (8.6) |
| Sex: number and % males | 149 | 93% | 141 | 90% |
| Primary site | | | | |
| Tongue | 53 | 33% | 54 | 34% |
| Tonsil | 79 | 49% | 76 | 49% |
| Velar | 15 | 9% | 17 | 11% |
| Other | 14 | 9% | 10 | 6% |
| T | | | | |
| 2 | 64[b] | 40% | 63 | 40% |
| 3 | 82 | 51% | 82 | 52% |
| 4 | 15 | 9% | 12 | 8% |
| N | | | | |
| 0 | 81 | 50% | 80 | 51% |
| 1 | 46 | 29% | 45 | 29% |
| 2 | 34 | 21% | 32 | 20% |
| Stage | | | | |
| 2 | 42 | 26% | 40 | 25% |
| 3 | 76 | 47% | 77 | 49% |
| 4 | 43 | 27% | 40 | 25% |
| ECOG performance status | | | | |
| 0 | 71 | 44% | 72 | 46% |
| 1 | 80 | 50% | 77 | 49% |
| 2 | 10 | 6% | 8 | 5% |

[a]SD: Standard deviation. [b]Includes one T1 patient included by error.

*© 2000 Cancer Research Campaign*

**Table 2** TNM distribution by treatment group

| Treatment group | T/N | $N_0$ | $N_1$ | $N_2$ | $N_{2a}$ | $N_{2b}$ | $N_{2c}$ | Total |
|---|---|---|---|---|---|---|---|---|
| No chemotherapy | | | | | | | | |
| | T1 | 0 | 0 | 1 | 0 | 0 | 0 | 1 |
| | T2 | 42 | 11 | 0 | 5 | 4 | 1 | 63 |
| | T3 | 34 | 31 | 0 | 9 | 5 | 3 | 82 |
| | T4 | 5 | 4 | 0 | 1 | 3 | 2 | 15 |
| | Total | 81 | 46 | 1 | 15 | 12 | 6 | 161 |
| Chemotherapy | | | | | | | | |
| | T2 | 40 | 12 | 1 | 6 | 4 | 0 | 63 |
| | T3 | 36 | 29 | 0 | 9 | 4 | 4 | 82 |
| | T4 | 4 | 4 | 0 | 1 | 0 | 3 | 12 |
| | Total | 80 | 45 | 1 | 16 | 8 | 7 | 157 |

**Table 3** Chemotherapy toxicity, maximum grade observed for each patient

| Type of toxicity | Grade | | | |
|---|---|---|---|---|
| | I | II | III | IV |
| Nausea, vomiting | 11 | 17 | 4 | 2 |
| Leucopenia | 8 | 17 | 10 | 2 |
| Thrombopenia | 2 | 6 | 3 | 1 |
| Mucositis | 4 | 8 | 5 | 3 |
| Cutaneous | 1 | 3 | 2 | 1 |
| Fatigue | 6 | 2 | 0 | 1 |
| Renal | 5 | 1 | 2 | 1 |
| Infection | 2 | 0 | 1 | 0 |

bucco-pharyngectomy had been planned, 2 only had neck dissection and radiotherapy to the primary and nodes.

### *Post-operative radiotherapy*

After chemotherapy and surgery, one patient with negative nodes and free surgical margins had no further treatment.

### *Radiotherapy alone*

7 patients had no radiotherapy: 6 were in the chemotherapy group (2 deaths including 1 from chemotoxicity, 2 by medical decision, 1 refusal, and 1 with a hypo-pharyngeal second primary) and 1 in the control group (death by suicide).

### *Chemotherapy*

In the chemotherapy arm, 97% of the patients received at least one cycle of chemotherapy, and 78% received the 3 planned cycles.

### *Toxicity of chemotherapy*

Among the 152 patients who received chemotherapy, 50% experienced toxicity (Table 3). Toxicity was grade 1 or 2 in 32%, grade 3 in 14% and grade 4 in 4%. These toxicities were mostly haematological (26%), digestive (22%), and mucositis (13%).

### *Response after chemotherapy*

56% of patients achieved an objective response, 20% a complete response and 36% a partial response (> 50%).

### Overall and event-free survival

The median duration of follow up was 5 years. At the time of the analysis 165 deaths had occurred, 73 in the chemotherapy group and 92 in the control group. 6 treatment-related deaths were observed, 3 per group.

A total of 40 patients never achieved a complete remission after treatment, 25 in the control group and 15 in the chemotherapy group. Event-free survival was considered 0 for these patients. Table 4 describes the type of first event by treatment group. Among the patients with death as the first event, half of them died of intercurrent causes, the cause of death was not available in 35% but they were free of recurrence and metastasis when last seen, and 16% died of treatment-related toxicity.

The overall survival curves and event-free survival curves are shown in Figures 1 and 2. The difference between the survival curves, adjusted on the initial therapy (radiotherapy alone or surgery plus radiotherapy) is statistically significant ($P$ = 0.03) for overall survival and in favour of the chemotherapy group (Table 5). No significant difference was found between the arms in event-free survival. Median survival is 5.1 years in the chemotherapy group versus 3.3 years in the control group.

When the results were stratified on local treatment (surgery + radiotherapy versus radiotherapy alone) no heterogeneity was demonstrated between strata in the effect of neoadjuvant chemotherapy on overall survival (Figure 3).

**Table 4** Type of first event by treatment group

| Type of first event | Treatment group | | |
|---|---|---|---|
| | No chemotherapy | Chemotherapy | Total |
| Loco-regional recurrence | 48 | 39 | 87 |
| Metastasis | 20 | 13 | 33 |
| Head and neck 2nd primary | 8 | 14 | 22 |
| Other second primary | 8 | 11 | 19 |
| Death | 20 | 15 | 35 |
| Total | 104 | 92 | 196 |

© 2000 Cancer Research Campaign

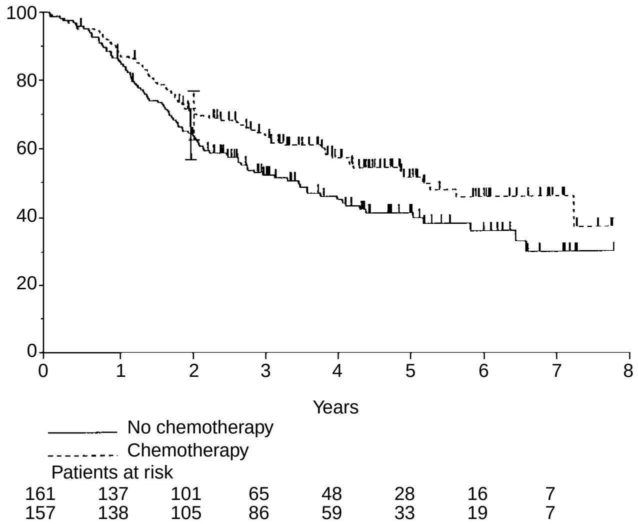

**Figure 1** Overall survival by treatment group

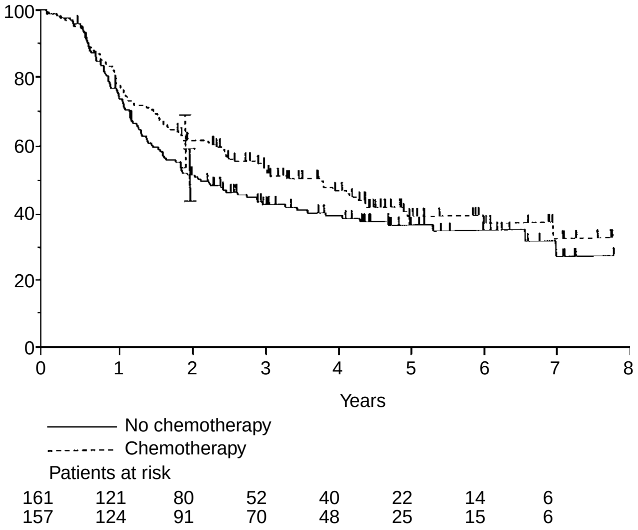

**Figure 2** Event-free survival by treatment group

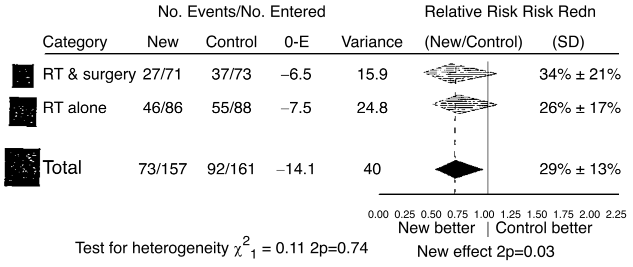

**Figure 3** Effect of treatment on survival according to loco-regional treatment

### Marginal hazards model

The results of the marginal hazards model are given in Table 6. There were no significant effects of chemotherapy on the risk of first local recurrence or head and neck second primary, on the risk of first metastasis, or on the risk of first second primary other than head and neck. As already shown with the logrank test, chemotherapy is associated with a significant reduction in the risk of death.

### DISCUSSION

This trial shows significantly better overall survival ($P$ = 0.03) in the neoadjuvant chemotherapy group than in the control group. The effect of neoadjuvant chemotherapy on event-free survival was smaller and of borderline significance ($P$ = 0.11).

The study was terminated after 6 years of accrual, before reaching the target sample size, because the investigators had lost interest in the evaluation of neoadjuvant chemotherapy. They were sufficiently convinced of its efficacy because it allowed organ preservation and because they believed that it was associated with longer survival owing to the observation of clinical and pathological responses after chemotherapy, considered predictive of longer survival. The decision to stop the trial was completely independent of the results which were not available at the time. It is therefore very unlikely that early termination is a cause of bias.

The chemotherapy effect we observed is consistent with the overall effect of chemotherapy demonstrated by Pignon et al (2000) in their meta-analysis, i.e. a 10% reduction in the risk of death. The effect of neoadjuvant cisplatin plus fluorouracil on survival in our trial (a hazard ratio for death of 0.71 with a 95% CI of 0.40–1.02) is in agreement with the significant benefit observed

**Table 5** Log-rank tests for survival and disease-free survival, adjusted for initial therapy (radiotherapy alone or surgery plus radiotherapy)

| | Number of deaths | | | | |
|---|---|---|---|---|---|
| **Chemotherapy** | **Observed** | **Expected** | **O-E** | **Var(O-E)** | ***P* value** |
| No | 92 | 78.0 | 14.0 | 40.7 | 0.03 |
| Yes | 73 | 87.0 | –14.0 | 40.7 | |

| | Number of events | | | | |
|---|---|---|---|---|---|
| **Chemotherapy** | **Observed** | **Expected** | **O-E** | **Var(O-E)** | ***P* value** |
| No | 104 | 92.9 | 11.1 | 48.7 | 0.11 |
| Yes | 92 | 103.1 | –11.1 | 48.7 | |

*© 2000 Cancer Research Campaign*

**Table 6** Relative risk for different types of event in the no chemotherapy group as compared to the chemotherapy group

| Type of event | Number of events | Relative risk | 95% CI | *P* value |
|---|---|---|---|---|
| Loco-regional recurrence or head neck second primary | 118 | 1.15 | 0.14–1.69 | NS |
| Metastasis | 54 | 1.36 | 0.79–2.34 | NS |
| Second primary other than head and neck | 25 | 1.23 | 0.55–2.75 | NS |
| Death | 165 | 1.39 | 1.03–1.88 | 0.04 |

NS = not significant.

with neoadjuvant cisplatin plus fluorouracil (hazard ratio 0.88, 95% CI 0.79–0.97) in the above-mentioned meta-analysis which includes our trial. Pignon et al (2000) found that the effect of a neoadjuvant regimen combining cisplatin and fluorouracil was significantly different ($P = 0.05$) from that of the other neoadjuvant regimens. These other regimens either did not contain cisplatin or were early studies of suboptimal cisplatin doses.

In conclusion, we observed better survival with induction chemotherapy combining cisplatin and fluorouracil than with no chemotherapy, in patients with oropharyngeal carcinoma. This is consistent with the results of Dr Pignon's meta-analysis. However, these results are not convincing enough to establish neoadjuvant chemotherapy with cisplatin and fluorouracil as a standard treatment, and new drugs will have to be compared to no chemotherapy in large randomized trials.

### ACKNOWLEDGEMENTS

We thank Cedric Mahé for the statistical analysis of marginal hazards, Laurence Désigné (secretariat of the Meta-Analysis of Chemotherapy in Head and Neck Cancer (MACHNC), and Rémi Lancar for statistical assistance and Lorna Saint-Ange for linguistic revision of the manuscript.

### REFERENCES

Calais G, Garand G, Beutter P and Le Floch O (1988) Advanced carcinoma of the oropharynx: the value of the induction chemotherapy before loco-regional treatment. A retrospective study of 138 cases. *Bull Cancer* **75** : 971–978

Decker DA, Drelichman A, Jacobs J, Hoschner J, Kinsie J, Loh JJ, Weaver A and Al-Sarraf M (1983) Adjuvant chemotherapy with cis diamminodichloroplatinum II and 120-hour infusion 5-fluorouracil in stage III and IV squamous cell carcinoma of the head and neck. *Cancer* **51**: 1353–1355

Ménégoz F and Chérié-Challine L (1998) Le cancer en France: incidence et mortalité. Paris: La documentation française

Pignon JP, Bourhis J, Domenge C and Désigné L, on behalf of the MACH-NC collaborative group (2000) Chemotherapy added to locoregional treatment for head and neck squamous cell carcinoma: three meta-analyses of updated individual data. *Lancet* **355**: 949–955

Schemper M and Smith TL (1996) A note on quantifying follow-up in studies of failure time. *Controlled Clinical Trials* **17**: 343–346

Weaver A, Fleming S, Vandenberg H, Drelichman A, Jacobs J, Kinsie J, Loh JJ and Al-Sarraf M (1985) Improved complete response rate and survival in advanced head and neck cancer after three-course induction therapy with 120-hour 5-FU infusion and Cisplatin. *Cancer* **55**: 1123–1128

Wei LJ, Lin Weissfeld. Wei LJ, Lin DY and Weissfield L (1989). Regression analysis of multivariate incomplete failure time data by modeling marginal distributions. *Journal of the American Statistical Association* **84**: 1065–1073

 *© 2000 Cancer Research Campaign*

## References

[PDF_00420] Calais G., Garand G., Beutter P., Le Floch O. (1988). Carcinomes avancés de l'oropharynx: valeur d'une chimiothérapie d'induction précédant le traitement locorégional. Etude retrospective de 138 cas.. Bull Cancer.

[PDF_00424] Decker D. A., Drelichman A., Jacobs J., Hoschner J., Kinzie J., Loh J. J., Weaver A., Al-Sarraf M. (1983). Adjuvant chemotherapy with cis-diamminodichloroplatinum II and 120-hour infusion 5-fluorouracil in Stage III and IV squamous cell carcinoma of the head and neck.. Cancer.

[PDF_00431] Pignon J. P., Bourhis J., Domenge C., Designé L. (2000). Chemotherapy added to locoregional treatment for head and neck squamous-cell carcinoma: three meta-analyses of updated individual data. MACH-NC Collaborative Group. Meta-Analysis of Chemotherapy on Head and Neck Cancer.. Lancet.

[PDF_00438] Rooney M., Kish J., Jacobs J., Kinzie J., Weaver A., Crissman J., Al-Sarraf M. (1985). Improved complete response rate and survival in advanced head and neck cancer after three-course induction therapy with 120-hour 5-FU infusion and cisplatin.. Cancer.

[PDF_00436] Schemper M., Smith T. L. (1996). A note on quantifying follow-up in studies of failure time.. Control Clin Trials.

